# AC-202, a highly effective fluorophore for the visualization of lipid droplets in green algae and diatoms

**DOI:** 10.1186/s13068-018-1117-9

**Published:** 2018-04-23

**Authors:** Seddik Harchouni, Ben Field, Benoît Menand

**Affiliations:** Aix Marseille Univ, CEA, CNRS, UMR7265 BVME, 13009 Marseille, France

**Keywords:** Algae, Fluorescent dye, Lipid droplets, BODIPY, Nile Red, AC-202, Chlamydomonas, Phaeodactylum

## Abstract

**Background:**

Lipid-specific live cell dyes are an important tool for the study of algal lipid metabolism, the monitoring of lipid production, and the identification of algal strains with high lipid yields. Nile Red and BODIPY have emerged as the principal dyes for these purposes. However, they suffer from a number of shortcomings including for specificity, penetration, interference from chlorophyll autofluorescence, and incompatibility with widely used genetically encoded reporters in the green and blue regions of the spectrum such as the green fluorescent protein and the red fluorescent protein.

**Results:**

We tested a new blue fluorescent dye, AC-202, in both the green algae *Chlamydomonas reinhardtii* and the pennate diatom *Phaeodactylum tricornutum*. We show that AC-202 is effective in both algae and that after minimal sample preparation, it can label lipid droplets induced by nitrogen starvation or by inhibitors of the TOR (target of rapamycin) kinase. We found that AC-202 benefits from a low background signal and is therefore more sensitive than BODIPY for semiquantitative in vivo fluorescence measurements. Finally, a co-staining experiment indicated that AC-202 can be used for multicolor imaging in combination with both red and green fluorophores.

**Conclusions:**

AC-202 is an alternative and highly effective fluorophore for algal research that resolves drawbacks encountered with other neutral lipid dyes. AC-202 can be used to rapidly and sensitively visualize lipid droplets, and will contribute to the identification of metabolic and signaling pathways involved in lipid droplet formation, monitoring lipid production, and in the development of screens for algal strains suitable for biofuel production.

**Electronic supplementary material:**

The online version of this article (10.1186/s13068-018-1117-9) contains supplementary material, which is available to authorized users.

## Background

Global economic activity relies on the consumption of large quantities of fossil fuels whose combustion releases greenhouse gases that are major contributors to climate change. Despite the growing adoption of renewable energy sources, fossil fuel use continues to increase [1]. The resulting continued growth in greenhouse gas emissions risks a scenario where global temperatures rise to levels that are predicted to have major negative impacts on society, the economy, and the environment [2]. Third-generation biofuels are fuels that are produced from microalgae and which therefore have the potential to help reduce net greenhouse gas emissions if adopted on a large scale. Microalgae are unicellular organisms that can fix CO_2_ during photosynthesis with a higher efficiency than plants [3, 4]. They can be cultured in closed photobioreactors or in open ponds directly exposed to sunlight. Notably, these methods of cultivation have the advantage of not competing with agriculture for land use. Microalgae are also one of the fastest growing photosynthetic organisms, and some are able to double their biomass in 24 h [5]. Under specific conditions, microalgae can also produce large quantities of neutral lipids, mainly in the form of triacylglycerol (TAG) that can readily be transformed into liquid fuels by transesterification. Lipid yields from microalgae can be more than tenfold greater than those from the best oilseed crops (oil palm) from the same land area [5]. Microalgae therefore have great potential for contributing to the replacement of fossil fuels with lower impact alternatives in the future. Despite the seductive advantages of microalgae, many bottlenecks limit the industrial production of third-generation biofuels [6]. One of these is the elucidation of the little-understood genetic and regulatory mechanisms controlling growth and the production of high-energy molecules and valuable byproducts. This understanding will in turn accelerate the domestication of microalgae by the selection and engineering of strains with robust growth and lipid production capabilities.

Under unfavorable growth conditions such as nitrogen depletion, TAG accumulates in lipid droplets where they are surrounded by a monolayer of polar lipids decorated by proteins [7]. The major functions of lipid droplets are to efficiently store energy and to provide acyl chains for membrane synthesis when environmental conditions again become favorable. The accumulation of lipids in microalgae can be detected using several methods including coherent Raman microscopy, gravimetry, thin-layer chromatography (TLC), high-performance liquid chromatography (HPLC), liquid chromatography coupled to mass spectrometry (LC–MS), gas chromatography coupled to mass spectrometry (GC–MS), and microscopy or flow cytometry using lipid-specific dyes [8]. The use of lipid-specific dyes is a rapid and cost-effective approach that allows the semiquantitative detection of neutral lipids in many organisms including microalgae [9]. Fluorescent lipid-specific dyes are particularly useful because they allow for the investigation of the size, number, localization, and dynamics of lipid droplets within living cells. The most commonly used lipid-specific fluorophores are Nile Red and BODIPY. These fluorophores have played an important role in recent advances in the understanding of lipid metabolism in microalgae [9]. However, despite their many advantages, these dyes do have certain drawbacks. Nile Red binds with different proteins depending on the polarity of its environment, and may therefore bias lipid detection [10–14]. The penetration of Nile Red into many microalgae is also difficult and often necessitates pretreatment of the cells that can affect their viability [15–22]. However, perhaps the most serious drawback of Nile Red is a broad emission profile that interferes in the green with fluorescent reporters such as GFP and in the red with chlorophyll autofluorescence in photosynthetic cells [9]. BODIPY 505/515 presents several advantages over Nile Red, because it is insensitive to the polarity of the environment, is specific to lipid droplets [11, 20], and shows good penetration characteristics [23]. BODIPY 505/515 is also more photostable than its predecessor BODIPY 493/503. However, the green fluorescence of BODIPY 505/515 (hereafter referred to as BODIPY) prevents its use with commonly used reporters such as GFP. Also, under certain conditions, a weak correlation between BODIPY fluorescence intensity and lipid content can be observed [24]. Alternatives to BODIPY have been developed for lipid staining, but these alternatives also fluoresce in the green region of the spectrum [25, 26]. Therefore, it is necessary to expand the palette of lipid-specific fluorescent dyes to take full advantage of modern multicolor cell-imaging techniques.

Recently, two new classes of blue fluorescent lipid-specific stains have been reported. Monodansylpentane (MDH) specifically labels lipid droplets with excitation in the violet and emission in the blue (420–480 nm) [27] and has been successfully used in plant cells [28]. However, MDH is a solvatochromatic dye that shows fluorescence in the red in aqueous environments [27]. Thus, care must be taken with the set-up of the fluorescence microscope, and multicolor imaging experiments could be complicated by this feature. Another class of lipid-specific blue fluorescent molecules (AC-201, AC-202, and AC-1041) was also recently reported in plants [29]. These markers are novel analogs of thalidomide (2,6-diisopropylphenyl-4/5-amino-substituted-4/5,6,7-trifluorophthalimides) that were originally developed as anticancer compounds [30]. These compounds show very low toxicity in plants cells [29]. The AC fluorophores are excited in the violet and emit in blue (410–490 nm) and are compatible for use with red and green genetically encoded reporters such as GFP and RFP, as well as with chlorophyll autofluorescence [29]. AC-201, AC-202, and AC-1041 have slightly different labeling and emission properties, and show excellent penetration and stability in several plant species. Currently, there have been no reports of the use of blue fluorescent lipid-specific dyes in algae, despite the intense study of lipid droplet biogenesis and turnover in these organisms.

In this study, we tested the potential of AC-202 as an alternative fluorophore for the visualization of lipid droplets in the green algae and in diatoms. We show that AC-202 specifically labels lipid droplets induced by nitrogen in the model green algae *Chlamydomonas reinhardtii* and the model diatom *Phaeodactylum tricornutum*. AC-202 showed excellent penetration and stability characteristics, and a markedly better signal-to-noise ratio than BODIPY. We also show that AC-202 can be used to label lipid droplets induced by treatment with an ATP-competitive inhibitor of TOR (target of rapamycin). Finally, we demonstrate that AC-202 can be used in combination with other fluorophores for multicolor imaging. Thus, AC-202 is a highly effective fluorophore for the visualization and quantification of lipid droplets, which is likely to be compatible with a broad range of microalgae.

## Methods

### Strains and cultivation conditions

*Chlamydomonas reinhardtii* CC4533 (wild type) was cultivated at 23 °C with shaking and under continuous light (50 µmol m^−2^ s^−1^) in standard Tris-acetate-phosphate (TAP) medium [31]. For nitrogen depletion, cells at OD_750_ = 0.5–0.7 were centrifuged at 3000×*g* 5 min and washed twice with TAP-N (TAP without NH_4_Cl). Pellets were then resuspended in TAP-N medium as in [32]. For TOR inhibition, AZD8055 (Chemdea, Ridgewood, USA) (dissolved in DMSO) was added to a final concentration of 2 µM in the medium at OD_750_ = 0.2–0.3, according to [33]. The final concentration of DMSO was 0.02%.

*Phaeodactylum tricornutum* was cultivated at 17 °C with shaking and under continuous light (50 µmol m^−2^ s^−1^) in Guillard medium f/2 + Si [34] that was made in filtered seawater (40 g L^−1^ of sea salts, Sigma S9883-500G). For nitrogen depletion, 6–7 mL of cells from a starter culture (OD_750_ = 0.5) were inoculated in 200 mL sodium nitrate free medium, or in control medium as described previously [35].

### Treatments with fluorophores

AC-202 (a gift from AVICOR Ltd, Hungary) and BODIPY™ 505/515 (ThermoFisher Scientific, Villebon-sur-Yvette, France) were dissolved in DMSO and added to 1 mL of cells at a final concentration of 10 µM. The final concentration of DMSO was 0.1%. Cells were then incubated in the dark for 10 min and washed twice with culture medium. After a final centrifugation, pellets were resuspended in culture medium and observed. MitoTracker™ Green FM (ThermoFisher Scientific) staining was performed as above, using 0.5 µM of the fluorophore.

### Microscopy

Brightfield and fluorescence images were obtained using an Axioimager M2 fitted with Axiocam HRc Camera (Carl Zeiss Microscopy, Marly le Roi, France). Fluorescence was visualized with HBO 100 mercury lamp and Plan-Apochromat 63×/1.40 Oil objective (Zeiss). Images were captured with AxioVision Rel 4.8 software. For fluorescence visualization, the filters were as follows: chlorophyll, excitation BP560/55, and emission BP645/75; AC-202, excitation BP365/50, and emission BP450/65; BODIPY, excitation BP470/40, and emission BP540/50. For quantification of fluorescence, cells were analyzed using an Apotome Z1 fitted with Axiocam MRM Camera (Zeiss). Fluorescence was visualized with HBO 100 mercury lamp Plan-Apochromat 63×/1.40 Oil objective (Zeiss). Images were captured using Zen 2012 Software. The filters were as follows: chlorophyll, excitation BP560/40, and emission BP630/75; AC-202, excitation G365 FT 395, and emission BP445/50 (Zeiss filter set 49); and BODIPY, excitation BP475/40, and emission BP530/50.

### Semi-quantitative analysis of the fluorescence

Fluorescence was quantified using ImageJ (version 1.45s, NIH). An outline was drawn around individual cells, and the area and the mean fluorescence were measured. Adjacent regions without cells were selected to measure the background fluorescence. The total corrected cellular fluorescence (TCCF) was then calculated using the following formula: TCCF = Integrated density − (Area of selected cell × Mean background fluorescence) as described by McCloy et al. [36]. Fluorescence intensity profiles were performed for single cells in ImageJ.

## Results

### AC-202 labels lipid droplets induced by nitrogen starvation in *C. reinhardtii*

**Fig. 1:**
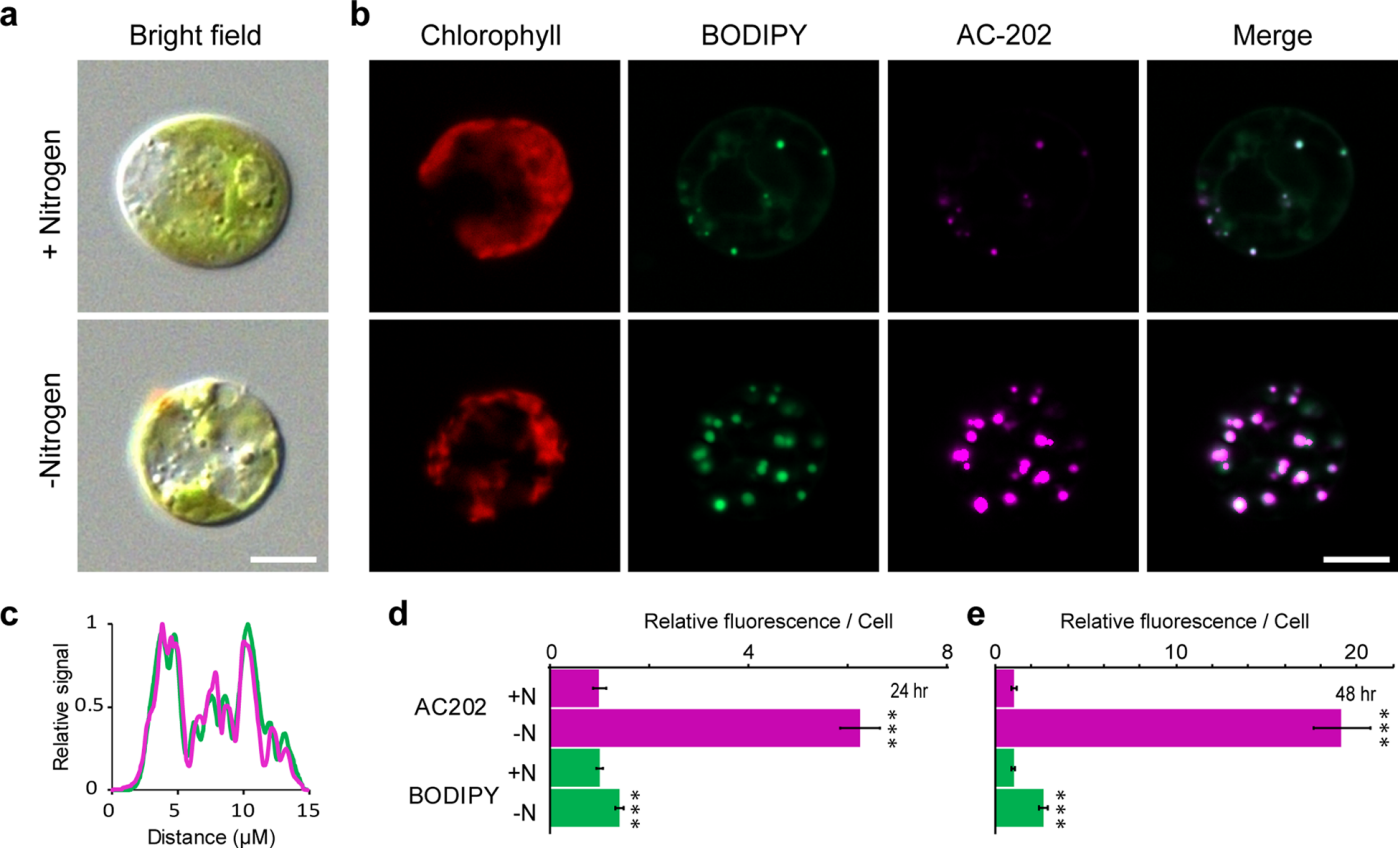
BODIPY and AC-202 stainings of lipid droplets in *Chlamydomonas reinhardtii* cells after nitrogen depletion. **a** Brightfield images of cells transferred to replete conditions (+Nitrogen, top) or media without nitrogen (−Nitrogen, bottom) after 48 h. **b** Fluorescence microscope images of cells transferred to replete conditions (+Nitrogen, top) or media without nitrogen (−Nitrogen, bottom) after 48 h and co-stained with BODIPY and AC-202. Single fluorescence channels and BODIPY + AC-202 fluorescence (Merge) are shown. **c** Normalized fluorescence profile plot of the nitrogen deprived cell in (**b**) showing the co-localization of BODIPY and AC-202 fluorescence. The *x*-axis represents the horizontal distance across the cell, and the *y*-axis the vertically averaged pixel intensity across the cell. **d**, **e** Total corrected cellular fluorescence was calculated for each fluorophore from cells grown under replete and nitrogen-starvation conditions after 24 h (**d**) and 48 h (**e**). Values are relative to the control condition (+Nitrogen). Averages shown, ± SE; ***P < 0.001, Students *t* test, *n* = 30; Scale bar, 5 µM

Nitrogen starvation is a well-known condition for the induction of TAG rich lipid droplets in algae [36, 37]. We therefore tested the fluorophore AC-202 using a simple staining procedure on *C. reinhardtii* cells transferred to growth media with or without nitrogen (Fig. 1). As a control, cells were co-stained with BODIPY, a previously characterized lipid-specific fluorophore. Cells grown without nitrogen accumulated large lipid droplets that were visible with both AC-202 and BODIPY at 48 h after transfer (Fig. 1b) (N.B. AC-202 is shown in mauve false color). The AC-202 and BODIPY signals appeared to co-localize completely, and no interference from the red channel for chlorophyll was observed. The co-localization of AC-202 and BODIPY was also confirmed by a fluorescence profile plot of a single nitrogen-starved cell (Fig. 1c). To objectively compare the two labeling methods, we quantified total corrected cellular fluorescence intensities per cell at 24 and 48 h post transfer (Fig. 1d, e). The quantification confirmed the increase in AC-202 and BODIPY fluorescence under nitrogen-starvation conditions relative to nonstarvation conditions. However, under nitrogen-starvation conditions, AC-202 showed a higher relative fluorescence signal than BODIPY at both 24 and 48 h post transfer (Fig. 1d, e). This can be explained by a much stronger signal-to-background ratio for AC-202 than for BODIPY, which appears to be due to diffuse cytoplasmic labeling by BODIPY under nonstarvation conditions (Fig. 1b, Additional file 1). Quantification of fluorescence in cells stained with each dye alone showed that the stronger signal-to-background ratio for AC-202 is not due to energy transfer from BODIPY to AC-202 in co-stained cells (Additional file 2). Together, these results indicate that AC-202 has a specificity for lipid droplets, which is very similar to BODIPY, has good penetration abilities, and can be used to visualize and quantify nitrogen-starvation-induced lipid droplets in *C. reinhardtii* with high sensitivity.

### AC-202 labels lipid droplets induced by the inhibition of TOR in *Chlamydomonas reinhardtii*

**Fig. 2:**
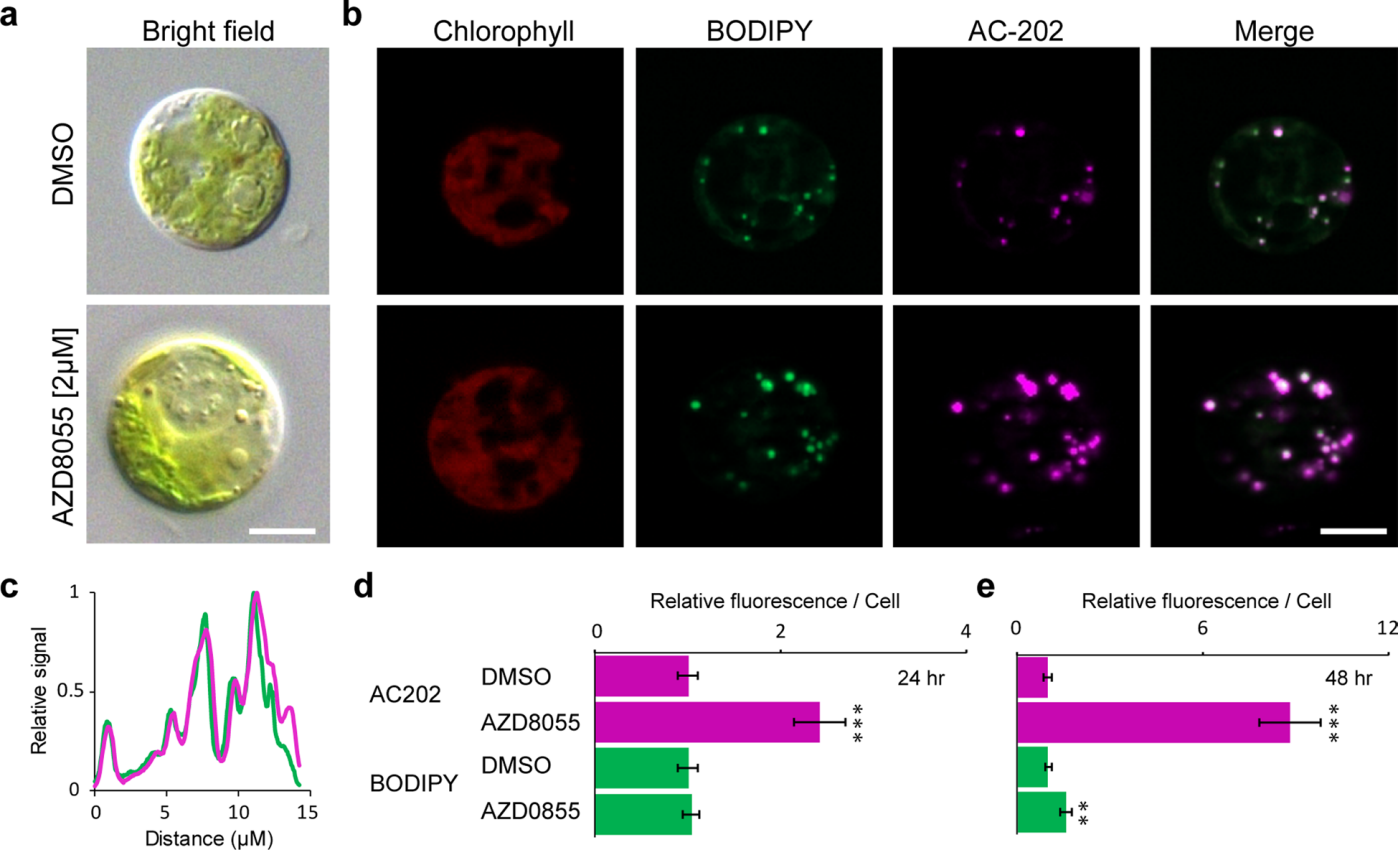
BODPIPY and AC-202 stainings of lipid droplets in *Chlamydomonas reinhardtii* cells in the presence of a TOR inhibitor. **a** Brightfield images of cells treated with DMSO (top) or a TOR inhibitor (AZD8055, bottom) after 48 h. **b** Fluorescence microscope images of cells treated with DMSO (top) or a TOR inhibitor (AZD8055, bottom) for 48 h and co-stained with BODIPY and AC-202. Single fluorescence channels and BODIPY + AC-202 fluorescence (Merge) are shown. **c** Normalized fluorescence profile plot of the AZD8055-treated cell in (**b**) showing the co-localization of BODIPY and AC-202 fluorescence. The *x*-axis represents the horizontal distance across the cell, and the *y*-axis the vertically averaged pixel intensity across the cell. **d**, **e** Total corrected cellular fluorescence was calculated for each fluorophore from cells grown under control (DMSO) or TOR inhibition conditions (AZD8055) after 24 h (**d**) and 48 h (**e**). Values are relative to the control condition (DMSO). Averages shown, ± SE; **P < 0.001, ***P < 0.001, Students *t*-test, *n* = 30; Scale bar, 5 µM

Inhibition of the eukaryotic TOR (target of rapamycin) kinase has been shown to induce lipid droplet formation in very diverse algae including the green alga *C. reinhardtii*, the red alga *Cyanidioschyzon merolae*, the Excavata *Euglena gracilis* and the diatom *Phaeodactylum tricornutum* [16, 33, 38, 39]. However, the TOR pathway remains little understood in these organisms compared to in plants or animals [40], and new tools are required for more in-depth investigations of TOR function. In order to test whether AC-202 can also be used to detect lipid droplets induced by the inhibition of TOR function, we treated *C. reinhardtii* cells with the specific ATP-competitive TOR inhibitor AZD8055 under conditions that were previously shown to induce lipid droplets [33] (Fig. 2). As expected, after 48 h, cells treated with AZD-8055 accumulated lipid droplets that were labeled with both BODIPY and AC-202 (Fig. 2b). The BODIPY and AC-202 signals co-localized completely in AZD8055-treated cells (Fig. 2b, c). Quantification of the fluorescence per cell showed that AC-202 staining allowed for the detection of a significant difference between nitrogen-starvation and control conditions 24 h after treatment. However, no significant difference between nitrogen-starvation and control conditions was observed with BODIPY staining at the same time point (Fig. 2d). After 48 h and in agreement with the microscopy images, we could observe a significant difference in fluorescence per cell for BODIPY, although this ratio was smaller than that of AC-202 (Fig. 2e).

### AC-202 can be used for multicolor fluorescence imaging

**Fig. 3:**
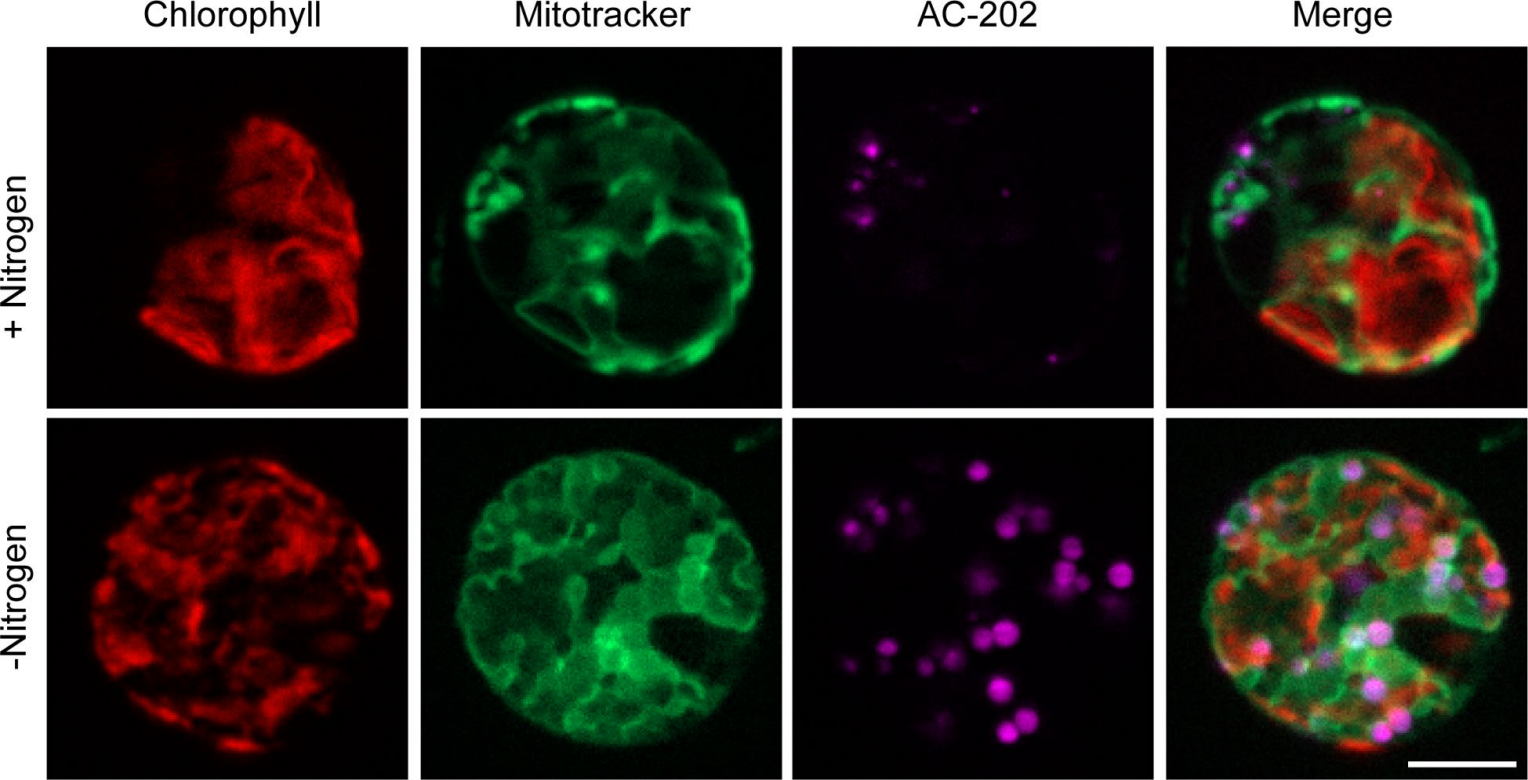
Multicolor imaging of *Chlamydomonas reinhardtii* cells after nitrogen depletion. Fluorescence microscope images of cells grown under replete conditions (+Nitrogen, top) or without nitrogen (−Nitrogen, bottom) and co-stained with MitoTracker and AC-202 48 h after transfer on to the nitrogen-depleted medium. AC-202 and MitoTracker can be used to simultaneously label lipid droplets and mitochondria, respectively. Scale bar, 5 µM

A problem encountered with BODIPY and Nile Red is that their fluorescence characteristics preclude their use with many known green and red fluorescent reporters such as GFP and RFP [41]. The spectral emission of AC-202 is from 410 to 490 nm, therefore it should be possible to use AC-202 simultaneously with other green and red reporters without interference. To test this idea, we stained nitrogen-starved cells with the green mitochondrial dye MitoTracker Green FM in combination with AC-202 (Fig. 3). We also visualized chlorophyll autofluorescence in the red channel. As before, AC-202 labeled the lipid droplets in the nitrogen-starved cells. MitoTracker Green stained diffuse cellular structures similar to those observed previously [42]. Merging the three channels showed that the structures identified in each channel are distinct (Fig. 3). This result indicates that AC-202 can be used for multicolor imaging in combination with green and red reporters and that it does not interfere with chlorophyll autofluorescence.

### AC-202 labels lipid droplets induced by nitrogen starvation in the diatom *Phaeodactylum tricornutum*

**Fig. 4:**
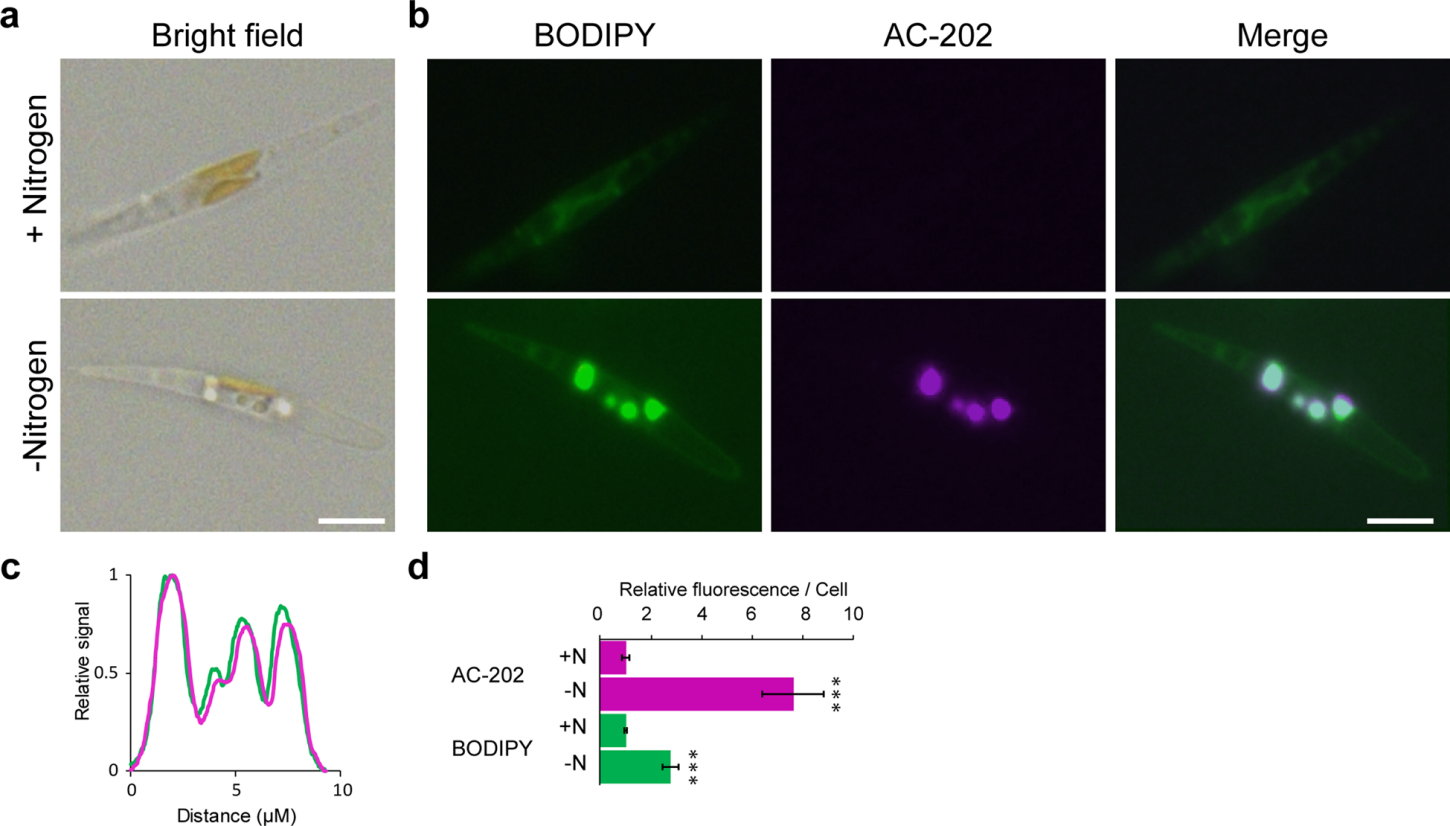
BODIPY and AC-202 staining of lipid droplets in *Phaeodactylum tricornutum* cells under nitrogen depletion. **a** Brightfield images of cells grown under replete conditions (+Nitrogen, top) or without nitrogen (−Nitrogen, bottom) after 72 h. **b** Fluorescence microscope images of cells grown under replete conditions (+Nitrogen, top) or without nitrogen (−Nitrogen, bottom) and co-stained with BODIPY and AC-202. **c** Normalized fluorescence profile plot of the cell in (**b**) showing the co-localization of BODIPY and AC-202 fluorescence. **d** Total corrected cellular fluorescence was calculated for each fluorophore from cells grown under replete and nitrogen-starvation conditions after 72 h. Values are relative to the control condition (+Nitrogen). Averages shown, ± SE; ***P < 0.001, Students *t*-test, *n* = 30; Scale bar, 5 µM

In order to test whether AC-202 has the potential to be used for the visualization of lipid droplets in a wide range of microalgae, we tested AC-202 on the distantly related chromalveolate model diatom *P. tricornutum*. Like many algae, *P. tricornutum* accumulates lipid droplets under stress conditions, including nitrogen starvation [35]. Therefore, we cultivated *P. tricornitum* in medium with or without nitrogen for 72 h and then co-stained the cells with AC-202 and BODIPY to visualize lipid droplet accumulation. As expected, lipid droplets accumulated in cells were grown without nitrogen and were readily visible with both AC-202 and BODIPY (Fig. 4b). The AC-202 and BODIPY fluorescence co-localized completely under nitrogen-starvation conditions (Fig. 4c). The quantification of fluorescence intensities per cell showed that the signal for both fluorophores increases significantly in response to nitrogen deprivation (Fig. 4d). However, as we also observed in *C. reinhardtii*, AC-202 showed a higher signal-to-background ratio than BODIPY. These results indicate that AC-202 can be used to visualize lipid droplets in the chromalveolate model diatom *P. tricornutum* with a higher sensitivity than BODIPY.

## Discussion

The use of lipid-specific dyes is a nondestructive, rapid, and semi-quantitative method to investigate the size, location, and dynamics of lipids droplets in living organisms. The two most used lipid dyes in microalgae are Nile Red and BODIPY [9]. Despite their important contribution to biofuel research on microalgae, these two dyes have some drawbacks including limited specificity to lipids (Nile Red), poor penetration into cells (Nile Red) and interference with chlorophyll autofluorescence (Nile Red) and green fluorophores (Nile Red and BODIPY). In this work, we tested the new blue fluorescent lipid dye AC-202 on the green algae *C. reinhardtii* and the model diatom *P. tricornutum*.

We show that AC-202 efficiently labels lipid droplets induced under nitrogen depletion in both *C. reinhardtii* and *P. tricornutum*, and in a manner very similar to BODIPY (Figs. 1, 4). AC-202 can also be used to label lipid droplets induced by treatment with TOR inhibitor in *C. reinhardtii* (Fig. 2). This indicates that AC-202 works well under different conditions of lipid droplet induction. Under all these conditions, AC-202 penetrated very well into algae cells indicating that it was not blocked by the membrane or cell wall of either algae, or by the silicified frustule of *P. tricornutum*. Quanti of the AC-202 signal indicated an increase of the total cellular fluorescence from 24 h of nitrogen depletion or treatment with the TOR inhibitor. Previous studies using direct GC–MS quantification show that an increase in TAG levels can be detected at 24 h under similar conditions, confirming the results that we obtained with AC-202 [16, 33, 38]. Likewise, N-depletion has been shown to cause a greater TAG increase than TOR inhibitor treatment, a phenomenon that we could also observe using AC-202 [16, 33]. Interestingly, under conditions of TOR inhibition, AC-202 fluorescence showed a quantitative difference at 24 h relative to the control while BODIPY did not. Along with the higher signal-to-background ratio, this result indicates that AC-202 is more sensitive than BODIPY, and that AC-202 fluorescence may also better reflect changes in TAG content. Indeed, a weak correlation between BODIPY fluorescence intensity and lipid content has previously been observed [24]. Thus, AC-202 may be a more useful tool for revealing subtle intracellular modifications in neutral lipid content. Finally, we have shown that in algae, and as predicted from its spectral emission characteristics, AC-202 does not interfere with red chlorophyll autofluorescence or with a green fluorescent dye (Fig. 3). These results underline the potential for combining AC-202 staining with both green and red fluorophores or genetically encoded reporters for multicolor fluorescence imaging in microalgae.

## Conclusions

Altogether, our data indicate that AC-202 is a robust and sensitive marker of lipid droplets that can be used for co-staining with red and green fluorophores in both green algae and diatoms. Thus, the use of AC-202 for labeling algal lipid droplets can resolve the major drawbacks that can be encountered with the fluorescent dyes that are currently used. We believe that AC-202 is therefore of great interest for the large community of researchers who are interested in the fundamental and applied aspects of neutral lipid accumulation in microalgae. For example, AC-202 could facilitate genetic or pharmacological approaches toward investigating the function of specific metabolic and signaling pathways involved in lipid droplet accumulation, be used for rapidly monitoring lipid production in an industrial setting, and has potential for use in the development of screens for algal suitable for biofuel production.

## Additional files

**Additional file 1.** BODIPY has a higher cytoplasmic background signal than AC-202 under nitrogen replete conditions. (**a**) Fluorescence microscope images of a cell (from Fig. 1b) under nitrogen replete conditions after 48 h. (**b**) A normalized fluorescence profile plot of the cell shows a higher cytoplasmic background for BODIPY compared to AC-202. Scale bar, 5 µM.

**Additional file 2.** Quantification of total corrected cellular fluorescence under single and co-staining conditions for BODIPY and AC-202. (**a**) Fluorescence microscope images of *C. reinhardtii* cells transferred to media without nitrogen after 48 h and stained with BODIPY and AC-202. (**b**) Total corrected cellular fluorescence was calculated for each fluorophore from cells grown under nitrogen starvation conditions after 48 h. Averages shown, ± SE; **P < 0.01, Student *t* test, n = 30 cells; Scale bar, 5 µM.

## Abbreviations

BODIPY: boron-dipyrromethene
TOR: target of rapamycin
TAG: triacylglycerol
GC-MS: gas chromatography coupled to mass spectrometry
GFP: green fluorescent protein
TAG: triacylglycerol
LC–MS: liquid chromatography coupled to mass spectrometry
RFP: red fluorescent protein
EGFP: enhanced green fluorescent protein
TAP: tris-acetate-phosphate
TLC: thin layer chromatography
