# Supplementary figures and images for: AC-202, a highly effective fluorophore for the visualization of lipid droplets in green algae and diatoms
Source: Biotechnol Biofuels. 2018 Apr 23;11:120. doi: 10.1186/s13068-018-1117-9 (PMC5913787; doi:10.1186/s13068-018-1117-9)

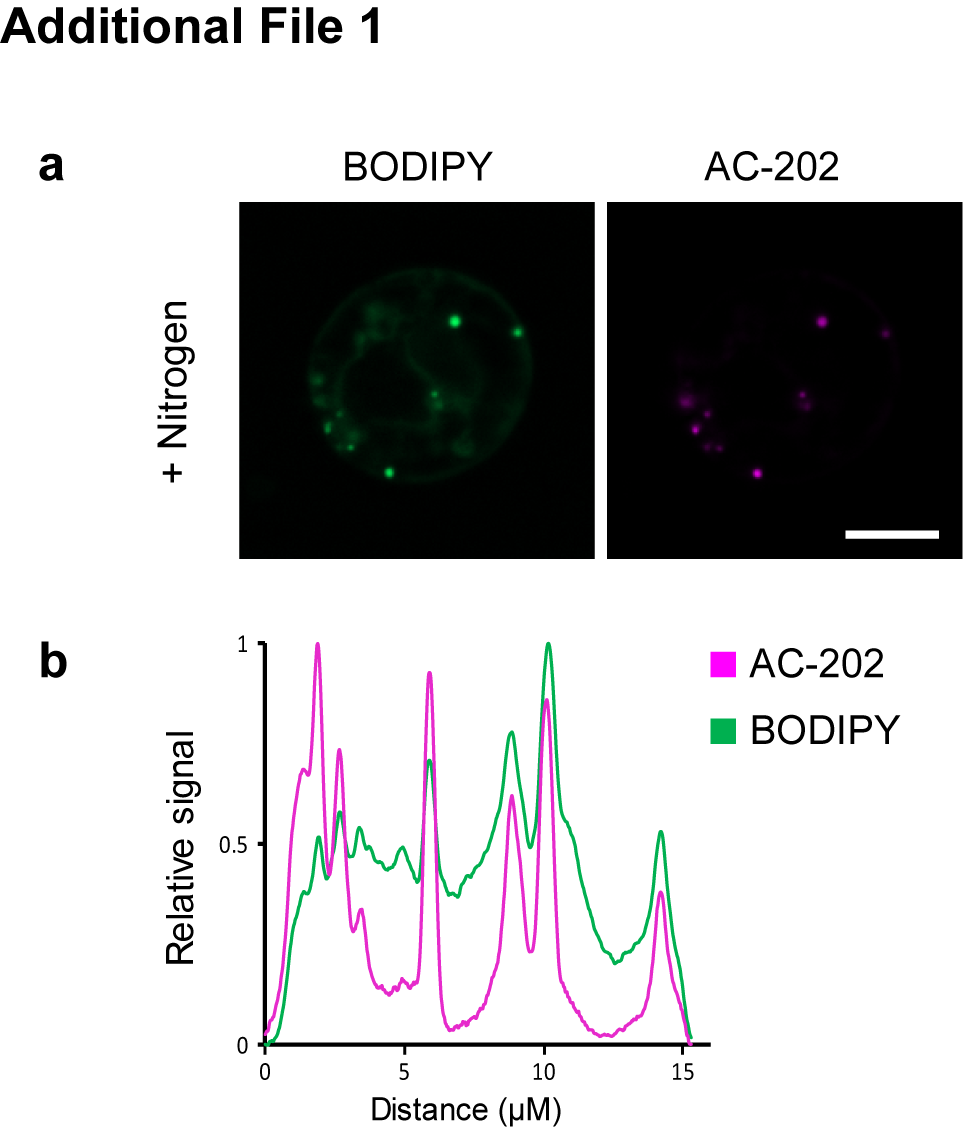

Supplement: Supplementary file 1 — Additional file 1. BODIPY has a higher cytoplasmic background signal than AC-202 under nitrogen replete conditions. (a) Fluorescence microscope images of a cell (from Fig. 1b) under nitrogen replete conditions after 48 h. (b) A normalized fluorescence profile plot of the cell shows a higher cytoplasmic background for BODIPY compared to AC-202. Scale bar, 5 µM. [file 13068_2018_1117_MOESM1_ESM.tif]

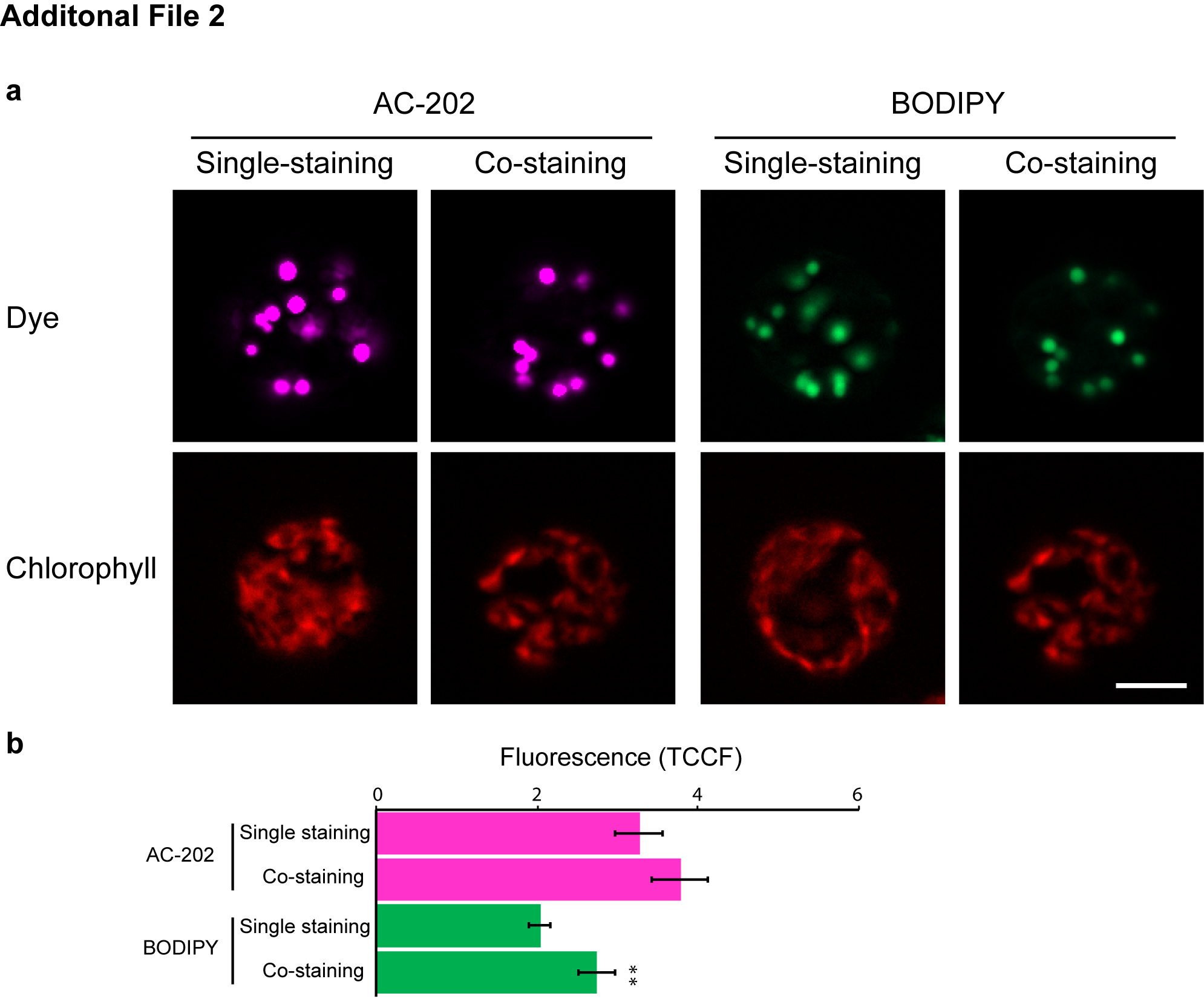

Supplement: Supplementary file 2 — Additional file 2. Quantification of total corrected cellular fluorescence under single and co-staining conditions for BODIPY and AC-202. (a) Fluorescence microscope images of C. reinhardtii cells transferred to media without nitrogen after 48 h and stained with BODIPY and AC-202. (b) Total corrected cellular fluorescence was calculated for each fluorophore from cells grown under nitrogen starvation conditions after 48 h. Averages shown, ± SE; **P < 0.01, Student t test, n = 30 cells; Scale bar, 5 µM. [file 13068_2018_1117_MOESM2_ESM.tif]
